# Respect in final-year student nurse–patient encounters – an interpretative phenomenological analysis

**DOI:** 10.1080/21642850.2014.918513

**Published:** 2014-05-21

**Authors:** Claudine Clucas, Hazel M. Chapman

**Affiliations:** ^a^ Psychology Department, University of Chester, Parkgate Road, ChesterCH1 4BJ, UK; ^b^ Faculty of Health and Social Care, University of Chester, Riverside Campus, Castle Drive, ChesterCH1 1SL, UK

**Keywords:** communication, health care, interpretative phenomenological analysis, professional norms, nurses

## Abstract

Very little is known regarding health-care professionals' understanding and experiences of respect towards patients. The study aimed to explore student nurses' understanding and experiences of respect in their encounters with patients. Semi-structured interviews were conducted with eight final-year student nurses with practice placements across different health-care trusts in the UK. Transcripts were analysed using interpretative phenomenological analysis (IPA). Three super-ordinate themes were identified: understanding of what it means to show respect, negotiating role expectations and personal attitudes in practice, and barriers related to the performance of the nursing role. The factors identified should be investigated further and addressed as they are likely to influence patients' experiences of feeling respected in nurse–patient interactions and subsequently their well-being and health-related behaviours.

## Introduction

A good nurse–patient relationship is needed for quality nursing care and is associated with lesser patient distress, greater disclosure of concerns and more active participation from patients in their care (Bowles, Mackintosh, & Torn, 2001; McCabe, 2004; Olson & Hanchett, 1997; Reid-Ponte, 1992). Nursing care influences patient perceptions of overall quality of care to a greater extent than physician care (Carey & Seibert, 1993), and perceptions of quality nursing care are largely determined by affective aspects of care (Attree, 2001). The quality of nurse–patient relationships merits research, since nurses are thought to spend more time with patients than other health-care professionals such as doctors, attend to more intimate aspects of care when the patient is particularly vulnerable and advocate on patients' behalf with other care providers, although this has become more difficult with changes in health-care delivery and increased patient demand (Castledine, 2008).

Respecting patients and their dignity is integral to the UK nursing code of practice (Chadwick, 2012; Francis, 2013), yet, little research has looked specifically at respect in health-care encounters. This is despite recent surveys, audits and a major inquiry suggesting respect is not optimal. Research based on national patient surveys has shown that being treated with respect and dignity is associated with patient satisfaction, adherence to advice, receiving optimal preventive care and seeking needed care (Beach et al., 2005; Blanchard & Lurie, 2004). Being treated with respect also has a positive impact on trust, illness perception and self-esteem (Clucas & St Claire, 2010); and respectful behaviours (e.g. taking time to talk, giving information, caring) are key to maintaining patient hope (Herth, 1990). Patients value respectful communication behaviours from nurses (McCabe, 2004; Shattell, 2004). Research is therefore needed to investigate nurses' and other health-care professionals' understanding of respect and their experiences of factors facilitating and hindering their respect towards patients.

Respect is an attitude directed towards someone based on his/her qualities that make him/her respect-worthy (Frei & Shaver, 2002), with cognitive, affective and behavioural components (Hendrick & Hendrick, 2006). Treating persons with respect means treating them as worthy/valuable human beings and valuing their humanity by being sensitive to their physical and psychological integrity (e.g. not condescending, not humiliating) and treating them as autonomous and rational human beings (e.g. respecting their point of view; Lalljee, Laham, & Tam, 2007). Important components of respectful behaviour in health-care contexts include treating patients as equals, taking them seriously (treating them as whole persons, paying attention to feelings and validating viewpoints), involving them in decisions, giving them information and treating them as important human beings (taking time with patients and being dedicated to their situation; Beach, Roter, Wang, Duggan, & Cooper, 2006; Clucas & St Claire, 2010; Purnell, 1999).

There is clearly an overlap between respect and the concepts of patient-centredness and person-centredness but the latter are difficult to explore because of their complexity and variation in application and they tend to lack conceptual clarity (Mead, Bower, & Hann, 2002; McCance, McCormack, & Dewing, 2011). Respect is a key underpinning aspect of the person-centred approach. A focus on respect helps integrate the dimensions of person-centredness around patients' experience of feeling worthy/valuable. This feeling might help explain positive associations between person-centredness and patient satisfaction and other positive patient outcomes since patients often experience feelings of vulnerability and inferiority in health-care settings (Crossley, 2000; Haskard, Zolnierek, & DiMatteo, 2009; Stewart, 1995). The study of respect can also draw on attitude theory, encouraging consideration of health-care providers' attitudes of respect towards patients, in addition to their attitudes towards behaving in a patient-centred/person-centred or respectful manner. According to attitude theory (Ajzen, 2005), it is possible to carry out specific patient-centred/respectful behaviours without holding a respectful attitude towards the patient, particularly if holding a positive attitude towards these behaviours. Patients are unlikely to feel respected unless they perceive the health-care professional as intending to show respect (Clucas & St Claire, 2011).

The present study used semi-structured interviews and interpretative phenomenological analysis (IPA) to explore the understanding and experiences of respect towards patients of final-year student nurses with practice placements across different UK health-care trusts. This is an important time in the socialisation of new nurses into the norms of the nursing profession and studying their experiences should help shed light on socialisation for patient respect in the nursing profession. Professional socialisation has been defined as

the complex process by which a person acquires the knowledge, skills, and sense of occupational identity that are characteristic of a member of that profession … and internaliz[es] the values and norms of the group into [his/her] own behaviour and self-conception. (Cohens, 1981, cited in du Toit, 1995, p. 168)

Professional socialisation takes place through education and the work environment and some aspects of professional socialisation acquired through education can be maintained in the work environment or abandoned in order to act professionally and adapt to the demands of the workplace (Lurie, 1981). An issue that has been raised is the conflict between the professional values acquired through nursing education and the ones embraced by clinical areas (Mackintosh, 2006). Although patient respect is integral to the UK nursing code of practice (Chadwick, 2012), some clinical areas/wards have been found to embrace less patient-centred and more task-focused norms where the emphasis is on completing physical tasks in a ritualistic manner (Maben, Latter, & Clark, 2006; Mackintosh, 2006; Mooney, 2007), with implications for respect shown to patients. IPA is a qualitative method that uses an idiographic and hermeneutic approach to phenomenological inquiry and provides a detailed account of experience, which makes it well suited to the aims of the study (Smith, Flowers, & Larkin, 2009).

## Methods

### Participants

Eight third-year adult nursing student nurses attending a university in the UK were recruited using a purposive sampling technique. About 120 nursing students received information about the study during lectures across university sites. Information sheets were given to those who showed an initial interest, who were then invited to contact the researcher for a verbal explanation. Participants (five diploma and three degree students) were recruited from four different education sites and had practice placements across four different UK health-care trusts. They were interviewed half way through their third year, on a programme where they had experienced continuing care in the first year, acute, critical and community care in their second year, and in their third year had experienced a further community placement and acute care placement. By this stage in the nursing programme, the students would have had interactions with patients, health-care assistants (HCAs) , nurses, doctors and other health-care professionals who are part of the health-care team but spend more time with registered nurses and other health-care professionals and less time with HCAs providing direct care to patients. Seven were female and one was male and their ages ranged between 18 and 60, which is typical of the university's student nurse population. Small samples are advised for IPA given its idiographic nature and the depth of the analysis (Reid, Flowers, & Larkin, 2005).

### Data collection

Ethical approval was granted by the Health & Social Care Faculty Research Ethics committee. Participants were reassured that their data would be anonymised and kept confidential and that their participation in the study would not affect their education or future. This was particularly important since the interviewer (the second author) was a lecturer on the programme. After informed consent was given, face-to-face semi-structured interviews took place in private at a convenient time and place for the participants in January and February 2012. Interviews lasted between 30 minutes to 1 hour and were digitally audio-recorded and transcribed verbatim. Each participant is referred to by a non-gender-specific pseudonym.

### Interview schedule

The interview schedule asked participants what respect for patients meant to them, expectations and behaviours of respect in practice, situations in which they have found it difficult to give respect to patients and factors that have facilitated or could facilitate their showing respect to patients and student nurses' attitudes of respect towards patients. The interview schedule was used flexibly to allow for exploration of responses and unanticipated themes.

### Analysis

Each individual case was subjected to an in-depth analysis. The authors first familiarised themselves with each transcript with a first reading noting interesting features and then engaged in phenomenological coding by applying line-by-line coding focusing on the participants' experiential concerns and cares (Smith et al., 2009). Participants' accounts were then interrogated by searching for patterns, contradictions and images employed to try to understand the messy sense-making of the participants, following which key issues/topics or themes were identified (Smith et al., 2009). The authors each worked on four interviews and then met to discuss the themes, checking the themes made sense and were backed by the data. Themes were then clustered into superordinate themes and compared across cases (Smith et al., 2009). The themes being reviewed by two researchers with different disciplinary perspectives (psychology and nursing) adds to the credibility of the analysis (Elliott, Fischer, & Rennie, 1999). However, participants' accounts could have been affected by their role as student nurses and by the study being conducted when nurses' behaviours were under public scrutiny (Cummings & Bennett, 2012).

## Results

Participants' accounts of what respect meant to them and of their experiences of respect in student nurse–patient encounters were analysed. Three key super-ordinate themes were drawn from the analysis: understanding of what it means to show respect, negotiating expectations in practice and personal attitudes and barriers related to the performance of the nursing role. A summary of the findings can be found in Table 1.

**Table 1.** Summary of the findings: brief description of themes.

Understanding of what it means to show respect	Negotiating role expectations and personal attitudes in practice	Barriers related to the performance of the nursing role
• Focus on describing the behaviours showing respect rather than the attitude of respect • Respecting and supporting patients’ autonomy • Showing emotional sensitivity and support, caring and building rapport • Not threatening patients’ psychological integrity	• Strong role expectations of respect (a) Embracing professional norms at a personal level but some feel do not belong to the nursing community (b) Not agreeing with disrespectful nursing practice – adherence to respect norms • Conflict between personal attitudes and role expectations (a) Beliefs regarding the consequences of respect and the need to respect patients (b) Difficulty negotiating personal feelings towards patients and role expectations • Strategies used to negotiate role expectations of respect and personal attitudes (a) Avoid conflict/disengage from emotionally difficult situations (b) Focusing on behaviours rather than attitudes (c) Denying vs. acknowledging negative feelings towards patients and using strategies to develop or maintain a respectful attitude	• Ward/organisational factors (a) Staffing shortages, heavy workload, emphasis on documentation, stress and shift handover as barriers to showing respect (b) Ward culture seeing spending time with patients as a health-care assistant role and/or characterised by ritualistic behaviours with a lack of patient input • Nature of the job (a) Seeing many patients leading to de-individualisation, invasive nature of the job and lack of opportunity to build rapport in A&E • Sense of perceived control

### Understanding of what it means to show respect

When asked what respect for patients meant to them, Chris explained respect for patients meant valuing patients by recognising they are persons with beliefs and preferences and Sam explained it meant recognising that patients are “human beings with opinions and feelings”. However, all other participants focused more specifically on describing the behaviours they believed were important in showing respect. Six participants, including Chris and Sam, saw respect in terms of respecting and supporting patients' autonomy, by giving them information to support them in making decisions and in particular by treating them as they would like to be treated by inquiring about and respecting their care preferences:

[I: asking for an example of respect towards patients] Jamie: … So rather than trying to undermine the way they do things, you let them do it the way that it suits them, and just accommodate as much as you can … allowing them to make the choices they would normally make, letting them stick to a routine that, that they've, you know, they're mature enough to know how they want to live.

Pat, however, focused less on patients' preferences: “Erm, I think fundamentally it means … to treat them as you wish, would wish to be treated” (Pat).

Participants also saw respect in terms of showing emotional sensitivity and support, caring and building rapport:

[I: asking about behaviours found helpful to show respect] Jamie: somebody was trying to insert this line anyway … she knew it was painful and quite often … it doesn't work. So I was there with her … when I held her hand she squeezed it, you know, and she kept squeezing it lots of times, so she really appreciated that, and … it never went in, she went through a lot of pain and then afterwards she was apologising for being upset … and I'm like ‘No! It's perfectly fine to be upset by something … I would be very upset if something hurt me like that … So I stayed in there with her for a bit, you know, as long as she felt like she shouldn't be crying…

While this was an important component of respect for Jamie, Pat and Billie, Ronnie's and Chris' accounts tended to embrace a more task-oriented view of care. Ronnie prioritised respect for autonomy over other components of respect, including caring for the patient's health outcomes: “You've got to respect whatever they want, even if you think it would be better otherwise, or if they did something differently it would make things better for themselves or for the staff” (Ronnie).

For five of the participants, showing respect meant not threatening patients' psychological integrity or not upsetting them, by respecting their privacy and maintaining their dignity, being professional (e.g. maintaining a calm front) and not being condescending or discriminatory. A key subtheme was respecting patients' privacy and maintaining their dignity:

Chris:** ** … ** **You've just got to give them all their care needs, what they need in a respectful manner, in like, dignity, privacy, because on the wards, you know, you've only got a curtain around, you're doing their daily routines, like the meds, wash** ** … ** **they've got to feel like they're being treated correct, because** ** … ** **they need, they just need to be respected.

Pat saw respect as treating patients equally and fairly: “To treat someone respectfully you have to treat them the way you would treat everybody else, which is completely equally and completely fairly** ** … ** **everybody's got to be the same” (Pat). This did not necessarily mean not giving patients the best individualised care as Pat later explained that treating patients differently to maintain the quality of their care might be the correct thing to do as long as it does not compromise the care of the other patients.

### Negotiating role expectations and personal attitudes in practice

#### Strong role expectations of respect

All participants strongly agreed that there was a reasonable expectation that nurses should behave with respect towards patients. In addition, they could lose their job or personal identification number (registration from the NMC) by acting disrespectfully. Pat explained that patients should be respected regardless of who they are or what they have done, as judging patients could affect their care:

Everybody, no matter who the person is or what they've done … they're all entitled to the same respect … We're here to treat people, not to judge them, that's the role of the courts, and the moment you start to make an assumption about somebody, you could miss information, you could treat them differently and you could compromise their care, and that … shouldn't happen. (Pat)

Six participants clearly embraced the professional norms at a personal level, seeing them as fitting with their personal values and beliefs. However, three of them also discussed not feeling they belonged to the nursing community because they were treated differently. Ronnie said:

I think I *will* feel I belong to the nursing community, at the moment as a student nurse, probably not. No-one really erm comes to you with information first, whereas as soon as you change your uniform for a blue erm *then* you are seen as part of the nursing team.

Alex felt excluded when he was not able to participate in discharged planning meetings despite having had a lot of input with a patient since this was not seen as appropriate: “ … I didn't feel valued then as the nurse, more of, as a student maybe, but not as a nurse as such, because I wasn't allowed to have my input in that session”.

Five participants (Sam, Alex, Jamie, Billie and Kelly) gave examples of disrespectful nursing practice by nurses, which they did not agree with, illustrating adherence to the norms defining the “good” nurse:

Billie: “A lady with dementia … was quite rude when shouting and calling people names, erm she wasn't doing what the nurse was asking her to and she just, she just spoke to her awful, like the tone of her voice she was using and the things that she was saying and I just thought, she's a patient and you can't treat her any different”.

These five participants also encountered instances of respectful practice (the other three participants only experienced good practice) and praised positive role models:

Jamie: “( … ) he was paying attention and, you know, being open to the patients and everything like that and … his behaviour and the sort of air he had around him was, was *really, really* professional and *really* nice and the patients really responded to …whenever he was working in the bay, it was really relaxed and err, I learnt, I learnt a lot from him”.

#### Conflict between personal attitudes and role expectations

Participants believed treating patients with respect empowered patients and made them feel more positive (Jamie) and led to better patient care since a personal bond is created, patient trust is gained (Pat, Chris) and patients cooperate and communicate more (Kelly, Pat and Ronnie). However, attitudes towards behaving with respect were not always positive. Pat believed that getting to know patients could make the nurse open to emotional hurt and Billie believed that spending more time with patients could lead to other jobs not getting done and other patients not getting seen. Also, Alex and Ronnie explained that they might not get respect in return. Despite this, they stressed the need to show respect as part of the role, highlighting the influence of role expectations of respect.

Kelly found it difficult to negotiate her personal feelings towards patients and the normative expectations of the role: “ … but sometimes … the way people speak to you and the way people, sometimes their respect isn't … But then again you know, you should treat them the same you know, shouldn't, *shouldn't* affect your respect with the patient”. Jamie explained that patients and the media expect nurses to not show feelings of irritation or frustration, although s/he saw this as almost impossible:

I think the views, really are getting a little bit impossible at the moment … from like a service user point of view, we're not supposed to ever be affected by anything, in all honesty, but from like … a personal side you, people, people will get irritated or stressed… we are going to feel that but I don't think that people think we should … .

#### Negotiating role expectations of respect and personal attitudes

The conflict between personal attitudes and normative role expectations was negotiated in different ways. Chris focused on normative expectations of behaving with respect rather than having a respectful attitude towards the patient:

 … if there's certain people who you don't get on with, you, you just, I don't know, I've learnt to do it and I just can't explain it, but you deal with them still in a respectful, professional manner, you still do it … . (Chris)

Chris also explains that s/he would avoid conflict with a patient with whom s/he does not get along by asking another colleague to take care of the patient. Avoiding conflict with patients appeared to be a strategy adopted by other health-care professionals on the ward to maintain a professional approach as Chris went on to explain:

 … .if [the patient] wasn't very nice to me or she was *really* rude to me, and I'd warn [colleagues], and if the patient was the same to them, then you'd get another colleague to go, and you'd find the one person who has a good rapport with that patient and you'd send them.

This strategy appears to tie in with a general belief that one way to fulfil the role expectations of respect is to disengage from emotionally difficult situations, in line with Jamie's earlier comment that nurses are not expected to have feelings of irritation or frustration.

Billie was reluctant to admit that negative behaviours of patients affected her/his respect for patients when her/his clinical skills were questioned: “It doesn't lessen my respect for them … it's probably more of a, a self-confidence aspect”. However, Billie admitted:

I said it *can* be harder to be respectful towards people when they are not treating *you* with respect, but I wouldn't say I would *ever* be the type of person to just not respect them altogether or just to have an opinion and willfully treat them different because of that … sometimes you can't help it like, like if somebody shouts at you … you're reaction or your, the face that you make because of that, they may see it as not respectful but that's just human nature … 

By acknowledging that negative non-verbal reactions could be interpreted by patients as signs of disrespect, Billie showed a deeper understanding of respect than Chris who believed that s/he could treat patients respectfully even if s/he did not get on with the patient.

Others admitted negative feelings towards or disagreements with certain patients but used varying strategies to maintain/develop a respectful attitude and behave respectfully in spite of these feelings. All participants felt their respect somewhat challenged when patients disrespected them, often by being rude or aggressive. Jamie and Kelly described initially negative feelings towards dementia patients because they could be aggressive or tested their patience but were able to gain understanding or empathy and change these. It was interesting to note that disrespectful attitudes towards dementia patients were prevalent in some wards. Sam and Billie witnessed disrespect for dementia patients but had more respectful attitudes themselves:

Other nurses would stick [a woman with dementia] in a bay of women who wouldn't speak to her and she'd be left on her own, or they'd leave her in the side room … .[staff would] ignor[e] the patients if they ask you a question, I've seen it a few times, especially when it's dementia patients asking questions, they'll just walk past. (Sam)

Jamie, Sam, Pat and Alex were also able to appreciate that patients often have valid reasons for behaving the way they do and their behaviour is often unintentional. Jamie stressed the need to step back from the situation to facilitate understanding of the patients' behaviour and not let the negative feelings affect his/her respect for his/her patients.

P … it's not that I lose respect for them … usually if you step back and have a look at the way that somebody might be acting towards you, erm, you can usually come to the root cause of why they're feeling like that and why they're displaying these emotions, or being disrespectful to you or being aggressive or anything like that … . (Jamie)

Still, it was interesting that Jamie did not always manage to gain empathy and sometimes withdrew emotional engagement:

 … sometimes if somebody's *really* rude or anything like that, at that immediate time you feel like your respect wants to completely withdraw, but when you walk away and think about it, you know, it's, it's like “Hmm, well if that's how you're going to be with me then forget it, you know, then I'll just go back to robot mode.”. (Jamie)

Sam and Billie explained that greater interaction with patients with mental-health issues and drug users can facilitate understanding of these patients and disconfirm negative stereotypes, although this was not the case for Ronnie who might have engaged at a more superficial level with patients: “it [the nursing course]'s opened my eyes to a lot of things but not changed my opinion of anyone really”. Also, Alex mentioned that constant abuse could lead to disrespect and Sam appeared to be struggling between his/her recognition that patients have good reasons for being rude or difficult and his/her negative feelings towards these patients.

[I: asking about attributes of service users that would lessen his/her respect for them]

Sam:  … patients who … even though they know they can't have treatment, they still ask for it… if they're rude, sometimes it's understandable, erm, once I got sworn at by a patient because … he needed a bedpan but he was adamant he was going to the toilet … when I explained ‘You need to have a bedpan, I know it's not the best thing in the world but it's the only way you can.’ And he swore at me. I wasn't impressed, but it's understandable, erm because nobody would want that… .

Pat and Ronnie also explained the need to respect patients' decisions/choices to which they are entitled and Pat saw worth in patients willing to risk their lives for their beliefs. This appeared to facilitate their respect for patients when patients consciously decided to act the way they did:

Pat: I have seen any number of very unhealthy, very obese people. Erm because of, my life style and the way I've been brought up, I disagree with their life choices not to look after themselves … . does not mean I res … that's their choice, they have made a conscious decision and I have to respect that decision … . I also respect that [Jehovah witnesses] hold a belief *so* firmly that they're willing to risk themselves for it, and *that* is another level of respect…

### Barriers related to the performance of the nursing role

Participants mentioned ward/organisational factors and the nature of the job as affecting their ability to show respect.

#### Ward/organisational factors

Five participants highlighted organisational factors in the form of the ward being short-staffed, being busy having to complete several tasks, the emphasis on documentation taking nurses away from direct patient care, stress and shift handover sometimes making it difficult to show respect:

Jamie: You know, even though I've got more time to spend with the patients than the trained nurses have, sometimes, you know, you might have three people that need something,  … and somebody might ask something when you're, when you're passing, like I'm, you know, “I can't really listen now, but is it okay if I come back in a minute?” or you sort of listen but not, you don't give them the sort of amount of time that you would normally

Billie:  … people are busy, shift handover, people go on breaks and they just forget … and then all of a sudden you'll realise that the curtains have been around this person for a long time … and you'll see that their bell is far away from them and they can't reach their bell and wonder how long they'd been there.

Alex and Kelly both saw stress resulting from pressures of the job as affecting respect levels. Alex commented: “[Nurses] seem to be very stressed at times which tends to erm get put on to the patients at times. ‘No I can't do this and I can't do that’, ‘can't you get up yourself’…’” Alex explained that workload pressures could make it difficult for nurses to take a step back when feeling stressed to not show disrespect. There were also concerns that the workload would worsen because of financial restrictions and staffing levels.

Billie also highlighted that nurses sometimes treat nursing as a series of tasks to be accomplished:

I think you should *choose* to be respectful rather than just treat it as a job and get on with it, and not really care about what the patient's thinking as long as the, the actual action is *done*, for some people I think they think that's alright, “Oh the jobs done, I'll walk away.”

The lack of patient input into aspects of care in line with a medical model and ritualistic behaviour was also highlighted by Alex and Jamie:

 … Ask them how they feel, they know themselves better than what we do. So ask them, “What do you want to do today?” You can make suggestions, “How about getting dressed?” … But I have seen, well you know, you're up at 6 o'clock today and you're getting out into your chair, why? (Alex)

For Alex, the culture where s/he worked also saw spending time with patients as a health-care assistant role, possibly because of nurses' documentation demands:

 … my mentor said I was working more as a health care assistant than a staff nurse … I was giving, in my opinion, the best standard of care I could and I wanted to *see* Mr Smith, and Mr Jones, and Mr Green because I wanted to know how they were, and I understood the nurse, who had so much input needed on the computer, erm entering the drugs and intravenous fluids … *but* physically I couldn't see how she could erm write a report on people that she hadn't seen.

Despite the sentiment that s/he could get in trouble for using a more personal and holistic approach to patient care, Alex felt confident enough to challenge staff, possibly due to being a mature student with many life experiences. Jamie also felt able to allow patients to take control over their care and highlighted her university course as an important source of support.

#### Nature of the job

Jamie thought seeing patients all the time can lead to losing sight of them as persons:

I think, being in the hospital and staying in there *all* the time, and then you're seeing patients in there all the time can almost sort of lead to that, that, that kind of patient … but, you have to, people are people, instead of looking at them as a patient, look at them as a person, that's what I'm trying to say. (Jamie)

Sam found it challenging to show respect when carrying out interventions that involve invading patients' personal space, especially in situations like A and E that do not provide enough opportunity to build a relationship with patients:

So I think especially with observations it can be challenging. The person doesn't like you in their personal space. *Yes* they have, yes the blood pressure can pass along the leads but still it's something in their personal space. So providing you get to know the patient, you're able to get a rapport with the patient it *can* help. But if you're on places like [ … ] A and E you don't know that patient … . (Sam)

Billie found it difficult to not get frustrated and show disrespect to less ill patients making demands when busy with a very ill patient:

if … there's one *really, really* ill patient, and then there's another patient maybe, I don't know, asking for a box of tissues and she's agitated because she hasn't got her tissues but *you're* looking after that really ill patient, it can be difficult to not just say, “Well, calm down, I'm looking after this patient”.

Despite barriers to showing respect, four participants (Alex, Jamie, Billie and Pat) emphasised that nurses were “ultimately” responsible for behaving respectfully and all generally reported feeling able to show respect. Chris and Sam insisted that they could always give respect to patients. However, they tended to focus on behaviours rather than attitudes of respect: “you may not have respect for a patient, but you mustn't show it … and if you haven't got *that* under control you could say things out of hand, which isn't professional … ” (Sam). Participants might also feel less able to show respect as they become qualified nurses: “while I'm a student I get to indulge myself in spending all that time with the patients and being there … but when I qualify you see the higher up you get, the further away from the patient you get” (Jamie). Alex explained that organisational barriers become more obvious when working as a qualified nurse. Billie also highlighted limited control over changing nurses' behaviours: “I think it's just difficult sometimes when you see other people not being respectful … even if you *did* do something, it doesn't mean that their behaviour is going to change … ”

## Discussion

Participants' understanding of respect tended to revolve around what it means to show respect as opposed to feelings involved. Their understanding of respectful behaviour was consistent with the literature on respect in health-care contexts (Beach et al., 2006; Clucas & St Claire, 2010; Purnell, 1999) or in more general contexts (Hendrick & Hendrick, 2006; Lalljee et al., 2007). However, the emotional healing aspect of respectful behaviour did not figure in all the participants' accounts. Several participants appeared to lack an integrated understanding of respectful behaviour as they placed emphasis on some components of respect to the detriment of others, which could have implications for patients' experiences of feeling respected as a result of the communication. Despite the importance of respect in health-care contexts, the present study is the first to provide an in-depth understanding of student nurses' (or nurses') understanding and experiences of respect with patients.

Participants all experienced strong expectations that nurses should behave with respect towards patients regardless of patients' behaviour or background. These had a powerful influence on participants' behaviour. Participants insisted that nurses should behave respectfully in the face of negative descriptive nursing team norms (instances of disrespectful practice from fellow nurses) or negative prescriptive nursing team norms (e.g. team culture not valuing spending time with patients), which they rejected. A focus on completing tasks in a set or prescribed way, accompanied by a lack of patient input into aspects of care, also characterised some of the wards but the participants insisted on involving patients in their care. Health-service reforms such as the introduction of skills mix in the 1980s to increase efficiency led to nurses undertaking more technical and medical-oriented tasks with HCAs taking on more of the essential care work (McKenna & Hasson, 2004). This could have resulted in technical care been perceived as higher status than caring work and spending “quality” time with patients, although the nursing mandate in the UK (and other Western countries) overtly espouses patient-centred holistic care that meets patients' psychological and physical needs (Maben, Latter, & Clark, 2007). However, a more medical and task-focused approach to care may be more characteristic of acute care as opposed to chronic care (Philpin, 1999).

Participants strongly valued patient-centred and holistic care towards the end of their educational period, in line with past literature (du Toit, 1995; Maben et al., 2007), although this was more evident in some accounts than others, and it is likely that several of the participants strongly valued taking care of people on entering into nursing (Mackintosh, 2006; Randle, 2003). In the third year of their programme, participants would have spent more time with registered nurses, developing their care management and supervision skills. Consequently, they would have become increasingly aware that direct care is associated with the role of the unqualified health-care assistant, while care management and administration are associated with the role of the registered nurse. This transitional period, at its height during the third year of the programme, may cause uncertainty of role, stress and feelings of loss but also a desire to hold onto the ideal of respect within a direct caring relationship.

Some studies have shown that student nurses can become less idealistic when exposed to clinical experiences with poor role models who do not value personal care and difficult emotional demands, to which they respond by hardening to avoid emotional burnout (Mackintosh, 2006; Randle, 2003). This was not found in our study, although some participants could have become somewhat less idealistic without being aware of it. Despite working in disrespectful environments, they had encountered positive as well as negative role models and had chosen to follow these positive role models who exemplified respectful care. One participant did express the concern that respect shown in getting to know patients could lead to emotional hurt but did not harden as a result. Previous literature has highlighted how students are able to critically select role models and “antimodels” (du Toit, 1995). This might have been facilitated by support from their course tutors or past life experiences. Also, it does not appear that they were in hostile working environments where they were treated negatively and/or bullied, which can lead to students adopting behaviours that go against their values such as devaluing patients or showing insensitivity to try to find acceptance (Hoel, Giga, & Davidson, 2007; Randle, 2003). Three participants mentioned that they did not feel that they belonged to the nursing community but this appeared to be due to not yet having a professional registration status and they appeared to be accepted and valued otherwise (Levett-Jones & Lathlean, 2008). Past literature has also highlighted that socialisation is an individual process to which students respond differently (Howkins & Ewens, 1999).

It is important to note that it might be harder to resist socialisation into disrespectful nursing practices once the student nurse starts working as a registered nurse. Newly qualified nurses who strongly valued patient-centred care on qualification were found to reconsider their “ideals” as they started practising as registered nurses when exposed to covert professional rules that went against patient-centred care, such as an emphasis on physical over psychological care and keeping an emotional distance from patients (Maben et al., 2006; 2007; Mooney, 2007). These covert professional norms might exercise more influence once student nurses practise as registered nurses because as qualified nurses, they feel a stronger sense of belonging to the nursing community (Terry, Hogg, & White, 1999), a possibility supported by the present study. The loss of the student role might also increase the desire to fit in with colleagues (Mooney, 2007). Newly qualified nurses might also be more fully aware than students of the covert norms or reconsider their role expectations given the stronger time and organisational pressures and stronger role demands (Maben & Clark, 1998). Trying to maintain the provision of good quality care can be exhausting and formal support is often lacking (Maben et al., 2006). The understanding that they cannot change the status quo, even as registered nurses, could also serve to crash nurses' ideals (Maben et al., 2006). Alternatively, covert professional rules do not exert more influence but qualified nurses are more reflective or more open to exploring their attitudes.

Participants were concerned that spending time with and respecting patients would become more difficult after nursing qualification, due to increased organisational demands and the stress of job pressures affecting their ability to show respect. Newly qualified nurses have been shown to experience the increase in responsibility and accountability related to drug administration, documentation and planning and prioritising of patient care as difficult and stressful (Higgins, Spencer, & Kane, 2010). Nurses in the UK face staff shortages, work overload and time pressures as well as a task-orientated approach to care that make person-centred care difficult, much of which is linked the global economic downturn and the need for delivery efficiency savings as well as increasing patient demand, a more open culture of accountability and meeting government targets (Maben et al., 2007; Wray, 2013). Moreover, the mismatch between newly qualified nurses' respectful care ideals and organisational demands could result in burnout and job dissatisfaction, with a negative impact on the care patients receive (Leiter, Harvie, & Frizzell, 1998).

Participants also had to negotiate role expectations and the sometimes negative feelings of frustration/irritation or upset experienced when patients were making unreasonable demands, were being “non-compliant” or disrespectful. This is in line with past research that has shown more negative attitudes towards patients considered demanding, non-conforming and disruptive (Breeze & Repper, 1998). Some participants were more willing than others to admit to “unprofessional” negative feelings and when they did often described strategies to maintain/develop a respectful attitude and behave respectfully, while others saw normative expectations of respect more in terms of behaving with respect rather than maintaining/developing a respectful attitude. There also appeared to be a general belief that one way to fulfil the role expectations of respect was to disengage from emotionally difficult situations, which led to strategies such as avoiding conflict with patients or disengaging emotionally to stay professional, which they might have acquired by observing other nurses or through contact with the media. Yet, this could compromise continuity and quality of care provided. Participants also differed in how aware they were that disrespect could be communicated unintentionally, such as through non-verbal behaviours. However, nurses' non-verbal behaviours shape the extent to which patients perceive nurses to be genuine (McCabe, 2004). How nurses negotiate their personal feelings/beliefs and the role expectations is important; patients are more likely to feel respected when the nurse is aware rather than denies her/his negative feelings and tries to maintain/develop a respectful attitude towards the patient. The study highlights some strategies used by student nurses to manage emotions and maintain an outward appearance of caring. This “emotional labour” of nursing is challenging and understanding it is necessary to reduce staff burnout and support quality patient care (Gray, 2009). Further research should investigate what accounts for such differences in the way personal feelings and role expectations are negotiated. Participants also witnessed disrespectful attitudes towards dementia patients, drug users and mental-health patients in the wards in which they worked but did not share these attitudes or had learnt to better understand these groups of patients and had developed more respectful attitudes.

A focus on behaviours rather than attitudes of respect is associated with a simplistic view of respect. This, a more superficial or task-oriented view of respect, access to a support system and/or student nurses experiencing less work constraints could help explain why participants reported feeling able to show respect despite organisational constraints and the invasive and task-focused nature of the job. Playing less of a role in essential care could also make it more difficult for nurses to show respect, having less opportunity to build rapport with patients.

The findings might not generalise to other final-year nursing students attending other universities in the UK, as the study's primary purpose was not to develop a representative but an in-depth account of final-year nursing students' experiences of respect. The present study has identified a number of factors that should be investigated further and addressed as they are likely to influence patients' experiences of feeling respected, including views of what respect means as well as what normative expectations of respect entail, the willingness (or lack of) to recognise negative feelings towards patients and have a strategy in place to deal with them, organisational and ward factors and challenges linked to the nature of the job. Future research should investigate registered nurses' experiences of respect and factors patients see as influencing nurses' behaviours of respect towards them.
